# Primary Catastrophic Antiphospholipid Syndrome With Multiple Infarcts in the Kidney, Liver, and Spleen in a Healthy Young Man: A Case Report

**DOI:** 10.7759/cureus.81621

**Published:** 2025-04-02

**Authors:** Ricardo Cid Puente, Erick R Melchor Bonilla, Alma P Huerta Alvarado, Paola D Aguirre Moreno

**Affiliations:** 1 Department of Immunology, School of Biological Sciences, Universidad Autónoma de Zacatecas, Zacatecas, MEX; 2 Department of Internal Medicine, Centenario Hospital Miguel Hidalgo, Aguascalientes, MEX

**Keywords:** anticardiolipin antibodies, antiphospholipid antibody, antiphospholipid antibody syndrome (aps), catastrophic antiphospholipid syndrome (caps), lupus anticoagulant antibodies

## Abstract

Catastrophic antiphospholipid syndrome (CAPS) is the most aggressive and severe form of antiphospholipid syndrome (APS). Due to its rarity and highly variable clinical presentation, diagnosing CAPS is extremely challenging, often leading to delayed treatment initiation and a high rate of mortality and morbidity. In this paper, we report the case of a 39-year-old male patient with no prior diagnosis of APS who initially presented with deep vein thrombosis in the left leg. The condition rapidly progressed to multiple thromboses in the lungs, liver, spleen, and left kidney. The diagnosis of CAPS was confirmed by the presence of lupus anticoagulant, and treatment was initiated 48 hours after symptom onset with high doses of steroids and anticoagulants. However, the patient developed multiple organ failure and died 72 hours after admission. This paper aims to highlight the rapid progression of CAPS, its fatal complications, and the critical importance of initiating treatment as quickly as possible, even before confirmation through laboratory or histopathological studies.

## Introduction

Antiphospholipid syndrome (APS) is an autoimmune disease of unknown etiology that may occur as a primary condition or secondarily associated with other autoimmune diseases such as systemic lupus erythematosus (SLE). Its main characteristic is the occurrence of thrombotic events in the presence of antiphospholipid antibodies (APLA) in serum [1]. Catastrophic antiphospholipid syndrome (CAPS) or Asherson's syndrome is the most severe form of APS [2]. It causes thrombosis in multiple organs (primarily kidneys, lungs, and brain, among others) and has a high mortality rate [3-5]. Its diagnosis is based on the 2002 classification criteria, which include the involvement of three or more organs in less than a week with the presence of APLA and histopathologic findings [6-7]. Treatment consists of the administration of anticoagulants, steroids, intravenous immunoglobulin (IVIG), and, in some cases, plasmapheresis or cyclophosphamide [8]. We report a case of a 39-year-old male who developed CAPS with multiple infarcts in the kidney, liver, and spleen with a fatal outcome. The importance of this case is to highlight the rapid progression of the disease, its clinical presentation, and the consequences of late diagnosis and treatment.

## Case presentation

A 39-year-old male presented to the emergency department with pain in the posterior aspect of the left leg, swelling, and claudication for three days. The patient had no relevant medical history and was in good health prior to this condition.

Upon admission, he presented with a fever of 39°C, sinus tachycardia at 122 bpm, blood pressure of 123/74 mmHg, a respiratory rate of 18 cpm, and an SpO₂ of 98%. Physical examination revealed tenderness in the posterior aspect of the mid-left leg, increased temperature and volume, no skin color changes, normal peripheral pulses, and a capillary refill time of two seconds.

Initial laboratory tests showed moderate thrombocytopenia (86,000/uL), a white blood cell count of 9.19 thousand/uL, and elevated acute phase reactants, including a C-reactive protein level of 344 mg/dL and a procalcitonin level of 6.2 ng/mL. Kidney function tests indicated a serum creatinine level of 1.5 mg/dL, an estimated glomerular filtration rate of 60 mL/min/1.73m², a blood urea nitrogen level of 27 mg/dL, and a urea level of 57 mg/dL. Liver function tests revealed an alanine aminotransferase level of 212 IU/L, an aspartate aminotransferase level of 196 IU/L, a lactate dehydrogenase level of 346 U/L, and an alkaline phosphatase level of 98 U/L.

Clotting tests were within normal limits, with a prothrombin time of 11.9 seconds, an international normalized ratio of 1.08, and a partial thromboplastin time of 34 seconds. A viral panel was negative for hepatitis C, hepatitis B, and HIV (Table 1).

**Table 1:** Laboratory findings during hospitalization NT-ProBNP: N-terminal pro-B-type natriuretic peptide, IgG: immunoglobulin G, IgM: immunoglobulin M

Laboratory tests	Admission	48 hours	Reference range
Hemoglobin (g/dL)	15.0	15.9	14.0-16.0
White blood count (1,000/uL)	9.19	30.0	4.5-11.0
Platelets (1,000/uL)	86.0	26.0	150.0-450.0
Serum creatinine (mg/dL)	1.5	3.5	0.6-1.2
Estimated glomerular filtration rate (ml/min/1.73m^2^)	60.0	22.0	>90.0
Blood urea nitrogen (mg/dL)	27.0	40.0	9.0-20.0
Aspartate aminotransferase (UI/L)	196.0	1101.0	17.0-59.0
Alanine aminotransferase (UI/L)	212.0	498.0	0.0-50.0
Conjugated bilirubin (mg/dL)	0.6	3.0	0.0-0.3
Unconjugated bilirubin (mg/dL)	0.7	0.8	0.0-1.1
Lactate dehydrogenase (U/L)	346.0	1693.0	120.0-246.0
Alkaline phosphatase (U/L)	98.0	296.0	38.0-126.0
C-reactive protein (mg/dL)	344.2	395.6	0.0-10.0
D-dimer (ng/mL)	2618.6	4738.8	<500.0
NT-ProBNP (pg/mL)	-	7840.0	0.0-125.0
Fibrinogen (mg/dL)	-	648.0	200.0-500.0
Lupus anticoagulant	-	2.4	0.0-1.2
anticardiolipin IgG	-	3.5	0.0-20.0
anticardiolipin IgM	-	1.0	0.0-20.0
Antinuclear antibodies	-	Negative	-

**Figure 1:**
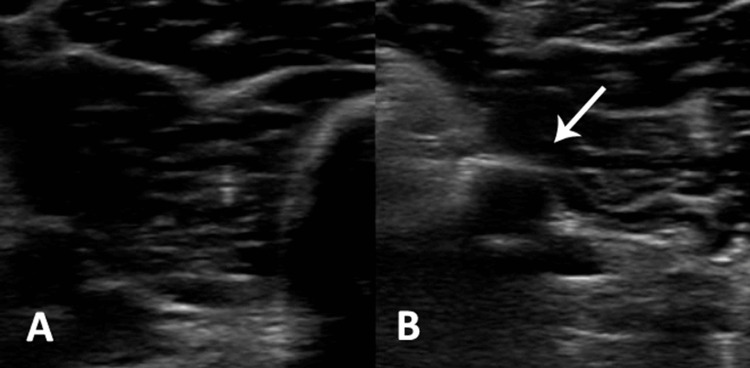
Ultrasound of the left pelvic limb In image (A), we can observe a completely dilated deep femoral vein. In image (B), on the other hand, we find a vein that does not collapse when pressure is applied (white arrow), which translates to the presence of a thrombus that is obstructing the vessel lumen.

A hepatic ultrasound revealed grade I hepatic steatosis, hepatomegaly of 176 mm, and splenomegaly of 142 mm. Due to suspicion of deep vein thrombosis (DVT) with moderate risk by Wells’ score, a serum D-dimer determination was requested, which was elevated 2,618 ng/mL; a Doppler ultrasound of the left pelvic limb confirmed the diagnosis of DVT with thrombosis of the deep femoral vein and posterior tibial veins (Figure 1).

**Figure 2:**
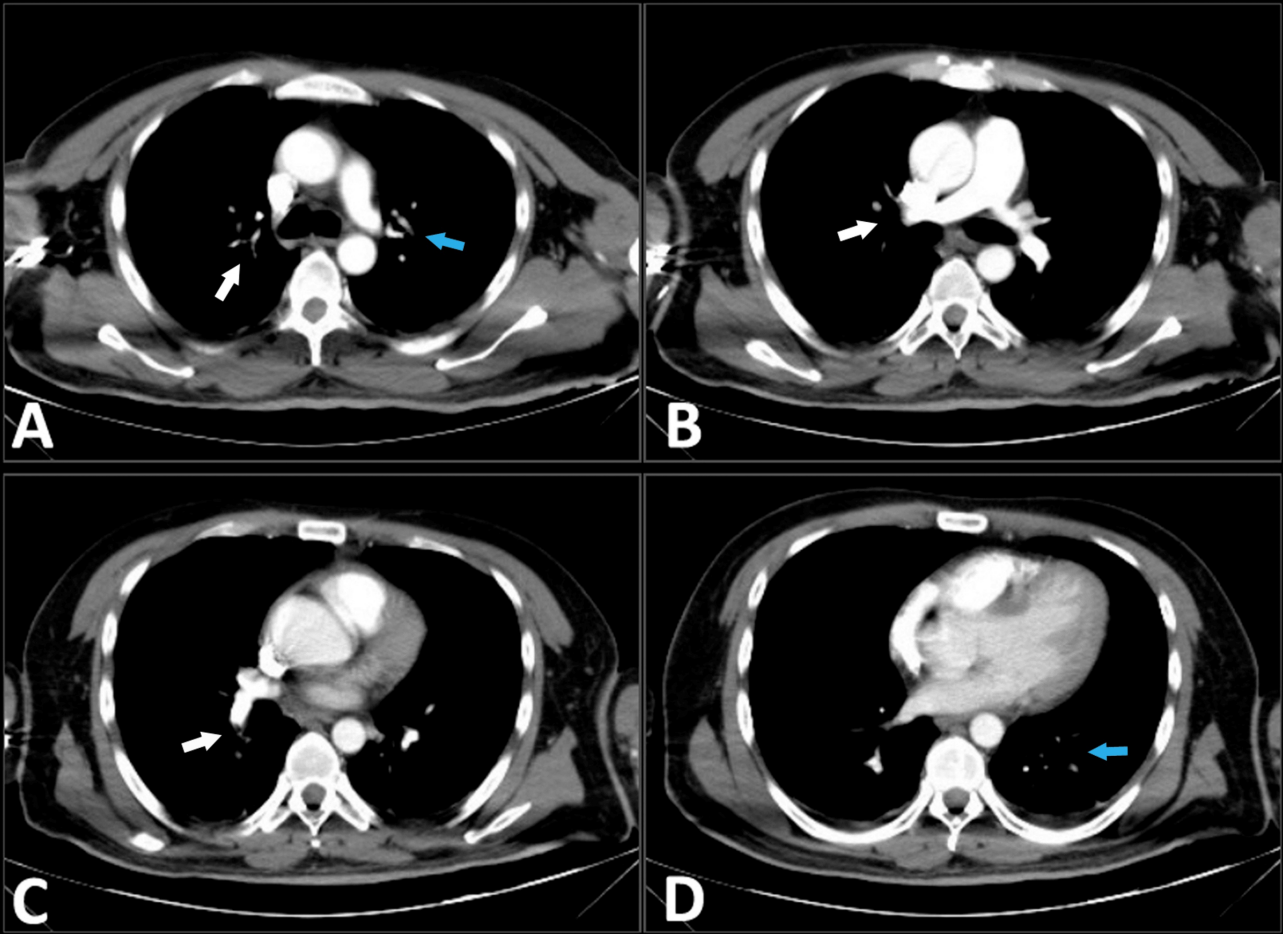
Contrast-enhanced CT pulmonary angiogram Filling defects (white arrows) are observed in the right lobar pulmonary artery and distal segmental arteries (A, B, and C). In the left hemithorax, filling defects (blue arrows) are observed in apical segmental arteries, as well as in some lower segmental arteries (A and D). CT: computed tomography

**Figure 3:**
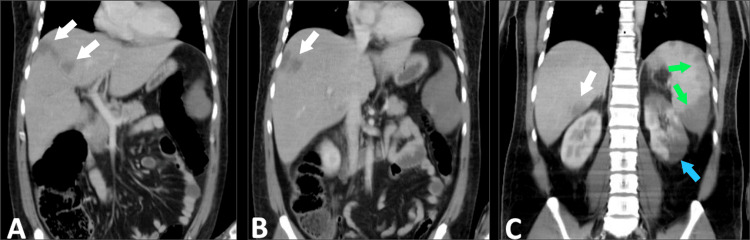
Contrast-enhanced abdominal CT White arrows show infarct areas in segments VIII (A and B) and III (C) of the liver. Green arrows indicate infarct areas in the spleen (C). The blue arrow shows an infarct area in the left kidney (C). CT: computed tomography

Management with enoxaparin 80 mg every 12 hours was initiated, and the patient was admitted to the hospital for monitoring. Forty-eight hours after admission, the patient developed tachycardia of 137 bpm, tachypnea of 30 cpm, and respiratory distress with decreased vesicular breath sounds in both hemithoraxes, along with generalized abdominal pain and abdominal distension. New laboratory tests showed a decrease in platelet count and worsening renal and hepatic function (Table 1). Due to suspicion of pulmonary embolism, a CT angiography was performed revealing pulmonary embolism of the right lobar and distal segmental pulmonary arteries and left apical segmental arteries, as well as thrombosis of the splenic artery, superior segmental artery of the left kidney, and segments III and VIII of the liver (Figures 2-3).

CAPS was suspected based on clinical presentation and imaging findings. Treatment was initiated with methylprednisolone 500 mg every 24 hours, intravenous unfractionated heparin (UFH) 25,000 IU at 13.5 ml/h, normal saline 0.9% at 80 ml/h, and buprenorphine 900 micrograms intravenously at 4.1 ml/h. Due to respiratory deterioration, advanced airway management was required. APLA determination was requested, revealing a positive lupus anticoagulant (LA) result (Table 1). Based on the classification criteria, a diagnosis of probable CAPS was made, as no histopathological study was performed to confirm microthrombosis. However, the involvement of three or more organs, rapid onset, and positive LA titers supported the diagnosis. Despite treatment initiation, the patient experienced cardiorespiratory arrest and died 72 hours after admission.

## Discussion

APS is a multisystem autoimmune disease characterized by thrombosis and recurrent pregnancy loss in the presence of APLA. It may occur as a primary condition or as a secondary condition associated with other autoimmune disorders such as SLE [1]. The disease is considered the most common acquired thrombophilia, and its clinical presentation varies between DVT and pregnancy morbidity (recurrent miscarriages, preeclampsia). However, approximately 1% of patients with APS will develop the most severe form known as CAPS or Asherson's syndrome. CAPS is characterized by the presence of multiple thromboses in three or more organs, often with rapid onset (less than a week), leading to multiple organ failure and elevated APLA titers [2-3]. Its mortality rate is high, reaching up to 50%, and is the first presentation of the syndrome in half of the cases [4]. Unlike APS, which affects large vessels in a single site, CAPS affects small vessels in multiple organs, including the kidneys (73%), lungs (60%), brain (56%), heart (50%), skin (45%), liver (34%), and less frequently the spleen, adrenal glands, and bone marrow [5]. In 2002, classification criteria were proposed with a sensitivity of 90.3% and specificity of 99.4%. A confirmed case meets all the following criteria: thrombosis in three or more organs, onset within less than seven days, small vessel micro-thrombosis by histopathology, and positive APLA at the time of diagnosis and 12 weeks after the event. A probable case is defined when only three of the four criteria are met [6-7].

In our case the differential diagnosis was made with other thrombotic microangiopathies such as immune thrombotic thrombocytopenic purpura (TTP) and hemolytic uremic syndrome (HUS). In the case of TTP, it was ruled out because clinically the patient did not present neurological symptoms or hemolytic anemia, and the peripheral blood smear did not show schistocytes; however, the determination of ADAMTS13 activity was not carried out. Similarly, HUS was excluded because it is a disease mainly seen in children with symptoms of gastrointestinal infections, which our patient did not present. Drug-induced microangiopathic syndromes and malignant hypertension were excluded as the patient denied taking any medication or drugs and maintained normal blood pressure levels during his hospitalization. Systemic infection was ruled out with blood cultures, which showed no growth. Finally, the patient met the criteria for probable CAPS as no histopathological study was performed, and lupus anticoagulant titers could not be corroborated 12 weeks after the onset of the symptoms [2].

According to the literature, the most common age of presentation of this disease is during the fourth decade of life, which matches the age of our patient. The most affected organs are the kidneys and lungs, both of which were involved in our case. Something to highlight in our patient's clinical presentation is the involvement of the liver and spleen, which are not frequent sites of thrombosis in CAPS, and the absence of thrombotic manifestations in the brain, one of the most affected organs [5].

CAPS treatment is multidisciplinary, and the first line of treatment is based on the so-called "triple therapy," which consists of the administration of high doses of systemic steroids, anticoagulation, and plasmapheresis or IVIG. This combination of drugs has been shown to be the most effective, with a survival rate of up to 73%. It consists of pulses of methylprednisolone 500-1,000 mg per day for three days, intravenous UFH followed by a transition to warfarin with a target international normalized ratio of 2-3 once the patient has stabilized, plasmapheresis performed daily until clinical improvement, and IVIG at a dose of 2 g/kg per day for two to five days. In cases of CAPS refractory to triple therapy, treatment is complicated and lacks high-quality evidence; nonetheless, the administration of second-line drugs is suggested. In general, the administration of intravenous cyclophosphamide 500-750 mg/m² is recommended in patients with SLE. In patients without SLE, monoclonal antibodies such as rituximab (anti-CD20) at a dose of 375 mg/m² per week for four weeks or eculizumab (prevents C5 complement cleavage/activation) at a dose of 900 mg per week for four weeks have been used; both have shown efficacy in the treatment of refractory CAPS [5,8].

Unfortunately, due to the lack of resources in our hospital, our patient only received double therapy with methylprednisolone and UFH, reducing the effectiveness of the treatment and increasing mortality.

Regarding the limitations of our case, the histopathological study could not be performed to classify the case as definitive CAPS, APLA titers could not be corroborated 12 weeks after the event, and the determination of anti-beta 2 glycoprotein antibodies was not carried out. The patient did not receive triple therapy; however, despite this, early treatment could have improved the patient's survival. If the diagnosis can’t be confirmed, we suggest that treatment should not be delayed.

## Conclusions

CAPS is a rare disease without a specific clinical picture that requires multidisciplinary management and high clinical suspicion for diagnosis. Despite early treatment, mortality remains elevated, and diagnosis is often not confirmed. Still, treatment should not be delayed if suspicion is high. Our patient had no relevant medical history, and the first manifestation was DVT that rapidly progressed to multiple infarcts because treatment was initiated 48 hours after the onset of symptoms. CAPS should be considered in young patients without significant medical history presenting with acute thrombotic events.
